# Molecular Evolution and Association of Natural Variation in *ZmARF31* with Low Phosphorus Tolerance in Maize

**DOI:** 10.3389/fpls.2016.01076

**Published:** 2016-07-21

**Authors:** Fengkai Wu, Zuoming Liu, Jie Xu, Shibin Gao, Haijian Lin, Ling Liu, Yaxi Liu, Yanli Lu

**Affiliations:** ^1^Maize Research Institute, Sichuan Agricultural University, WenjiangChina; ^2^Key Laboratory of Biology and Genetic Improvement of Maize in Southwest Region, Ministry of Agriculture, WenjiangChina; ^3^Triticeae Research Institute, Sichuan Agricultural University, WenjiangChina

**Keywords:** favorable allele, low phosphorus tolerance, maize (*Zea mays*), natural variation, root morphology, *ZmARF31*

## Abstract

Low-phosphorus (P) stress is one of the major factors constraining plant growth and yield. Improving plant tolerance to P starvation through molecular breeding is an efficient alternative to increase grain production. In the study, 331 diverse maize inbreds were used to detect nucleotide diversity and favorable alleles of *ZmARF31*, which plays a key role in low P responses and root architecture regulation. Significant phenotypic variation was found in each of 11 tested traits under both P and no-P treatments, and 30 single nucleotide polymorphisms (SNPs) and 14 insertion–deletions (InDels) were detected in *ZmARF31* among the 331 maize inbreds. The 5′-untranslated region (UTR) of *ZmARF31* showed a small linkage disequilibrium (LD) block under significant purifying selection, whereas the 3′-UTR showed the most abundant diversity and a larger LD block. Thirty, fourteen, and nine natural variations were identified in *ZmARF31* that were associated with P-deficiency-tolerance traits (*P* ≤ 0.01) by using the general linear model (GLM), GLM incorporated with population structure, and mixed linear model, respectively. Four SNPs were significantly associated with the total dry weight (TDW) in the three models, of which SNPs S410 and S462 were located in a complete LD block. A further verification conducted in a recombinant inbred line population revealed that favorable allele G/G of non-synonymous mutation S410 and favorable allele with a 38 bp insertion of InDel S1442 exhibited positive genetic effects on the TDW and total root tips, respectively. Expression analysis further confirmed that *ZmARF31* was highly expressed in the roots of low-P-tolerant inbred 178. The protein encoded by *ZmARF31* was located both in the nucleus and cytoplasm. Haplotypes carrying more favorable alleles showed a greater effect on phenotypic variation than single loci. Such haplotypes should be helpful to develop valuable genetic markers and perform maize molecular breeding.

## Introduction

In plants, phosphorus (P) is a critical macronutrient and plays an important role in various basic biological functions such as energy generation, glycolysis, nucleic acid synthesis, enzyme activation/inactivation, redox reactions, signaling, and carbon metabolism (Rausch and Bucher, 2002; Cordell et al., 2009; Shen et al., 2011). Low availability in soil and inadequate supply of P are major constraints for crop production. Improving plant tolerance to P starvation is an efficient alternative to increase the total grain production. Therefore, elucidating P starvation responses in plants at the molecular level is useful for developing improved genotypes that might perform well under P deficient conditions.

Many studies have been attempted to better understand the developmental and genetic mechanisms underlying P deficiency in plants. To cope with P limitation, plants have evolved complex adaptive mechanisms to enhance the uptake and utilization of P, including morphological, physiological, biochemical, and molecular modifications (Raghothama and Karthikeyan, 2005; Calderon-Vazquez et al., 2008; Peret et al., 2011). At present, some studies have been focused on the complex network of associated strategies and regulatory genes involved in phosphate uptake, remobilization, and metabolism (Lopez-Arredondo et al., 2014). P starvation-induced genes that regulate root morphogenesis have been identified, such as *AtTIR1* (Perez-Torres et al., 2008), *OsPHR*2 (Wu et al., 2013), *ZmPTF1* (Li et al., 2011), and *AtARF*s (Okushima et al., 2005; Gutierrez et al., 2009). The auxin response factor (*ARF*), one of the transcription factors (TFs), plays a key role in low P responses and phenotypic variation by altering gene expression and function (Calderon-Vazquez et al., 2011). The knockout of *OsARF16* led to primary roots, lateral roots, and root hair losing sensitivity to low-P and auxin response, which provided a novel evidence of a linkage between auxin and low-P responses in rice (Shen et al., 2012). In *Arabidopsis*, *ARF*s involved in the regulation of root architecture are also the most important regulators of downstream starvation-induced genes (Okushima et al., 2005, 2007; Gutierrez et al., 2009). The *ARF* gene family has been shown to include numerous members, with 23, 25, and 31 members in *Arabidopsis*, rice, and maize, respectively (Xing et al., 2011). Traditional genetic approaches have verified the function of *ARF* genes in plant growth and development. Four *OsARF* genes were positively associated with plant height and tillering ability in *Oryza sativa* (Liu et al., 2012). Target-gene analysis of *arf7–arf19* transgenic plants revealed that ARFs regulated lateral root formation via the direct activation of lateral organ boundaries domain/asymmetric leaves (*LBD/ASL*s) in *Arabidopsis* (Okushima et al., 2007). The conserved domains of *ZmARF31* in maize are highly similar to those of *AtARF19* in *Arabidopsis*, with known function in root growth and development (Okushima et al., 2007).

The findings of such studies provide more information to accelerate molecular breeding for selecting P-deficiency-tolerance traits by using useful molecular markers. Based on high density of genetic polymorphisms and linkage disequilibrium (LD), association analysis provides a novel approach for dissecting complex trait loci in maize (Liu et al., 2013). Maize shows an abundant genetic diversity and rapid LD decay. On average, one single nucleotide polymorphisms (SNPs) or insertion–deletion (InDel) exists every 44 bp across the 10 maize chromosomes (Gore et al., 2009), and intragenic LD declines within 100–200 bp along chromosome 1 of maize (Tenaillon et al., 2001). This rapid LD decay probably reflects high recombination rate within genes (Remington et al., 2001), along with high genetic polymorphism. Over 1.06 million SNPs obtained from RNA sequencing and DNA array were used to conduct a genome-wide association analysis to elucidate the genetic architecture of maize oil biosynthesis (Li et al., 2013) and 17 agronomic traits in a diverse maize population (Yang et al., 2014). Compared with linkage mapping, association mapping offers more advantages, such as time and cost effectiveness and increased mapping resolution; more importantly, this method can be used to investigate simultaneously greater allele numbers (Yu and Buckler, 2006). The candidate gene-based association analysis aims to improve the odds of identifying the most important alleles, including SNPs and InDel, which might be the potential molecular markers contributing to complex traits (Yan et al., 2011). A candidate-gene association study indicated that six natural *GmACP1* polymorphisms explained 33% of the phenotypic variation. The favorable alleles and haplotypes of *GmACP1* are associated with increased transcript expression correlated with higher enzyme activity (Zhang D. et al., 2014). The association between the nucleic acid variations of each dehydration responsive element binding protein (DREB) family gene with drought tolerance was evaluated using a diverse maize population, and a significant genetic variation in the gene promoter of *ZmDREB2.7* was identified to be associated with drought tolerance (Liu et al., 2013). With high-efficiency molecular marker analysis and advanced genetic mapping techniques, elucidating the natural variation within genes and determining the genetic basis of the maize root system in response to low-P stress have become possible. This study aimed to (1) detect favorable alleles and haplotypes within *ZmARF31* in response to the low-P stress and molecular evolution in maize germplasm and (2) further validate gene expression in low-P-tolerant and low-P-sensitive inbreds and the genetic effect of favorable alleles on P-deficiency-tolerance traits in a recombinant inbred line (RIL) population. Molecular markers designed based on favorable allelic variations and haplotypes are useful in molecular breeding to improve maize tolerance to P deficiency.

## Materials and Methods

### Plant Materials and Phenotyping

In total, 331 diverse core maize inbred lines (131 temperate and 200 tropical/subtropical lines), selected from different heterotic groups, stiff stalk, non-stiff stalk, and tropical or subtropical group (Xu et al., 2009; Yang et al., 2011; Li et al., 2012), were used to construct a maize association panel (Supplementary Table S1). The plant materials were planted in plastic pots filled with river sand in the greenhouse of Sichuan Agricultural University, China (Ya’an, E 103°01′, N 29°54′) during 2010 and 2012 (Zhang L. et al., 2014), respectively, to avoid rain and other influencing factors. All maize inbred lines were evaluated under P-applied (PA) and non-P-applied (NPA) conditions in a randomized complete block design with two replicates. For each replication, eight seeds were sowed in one pot per line and then the seedlings were thinned to five. For the PA treatment, normal nutrient solution modified from Hoagland and Arnon (1950) was applied, while no NH_4_H_2_PO_4_ was added to the solution for NPA treatment. The plants were watered once every 3 days with 300 mL of the solution per pot. The P-deficiency-tolerance traits were evaluated at the five-leaf seedling stage, i.e., 25 days after germination.

The WinRhizo Pro 2008a image analysis system (Regent Instruments Inc., Quebec, QC, Canada) equipped with a professional scanner (Epson XL 1000; Japan) was used to analyze the root morphology under PA and NPA conditions, such as total root length, average root diameter (RD), total root volume, total root tips (RT), and total root fork. The other characteristics measured included number of leaves (NL), length of the longest root (LLR), total dry weight (TDW), root dry weight, shoot dry weight, and root to shoot ratio. The plant material was separated into shoot and root, stored in paper bags, and heated at 105°C for 30 min to kill the living cells, and then dried at 75°C until constant mass was obtained. The field experiment conducted in 2012 has been reported previously (Zhang L. et al., 2014). Descriptive statistics, including variation range, mean value, standard deviation, and analysis of variance (ANOVA) with univariate general linear models (GLMs) was computed using SPSS Statistics 17.0 (SPSS Statistics, 2008).

A RIL population (178 × 9782) containing 196 lines generated using low-P-tolerant inbred (178) and low-P-sensitive inbred (9782) was used for the validation of significant association sites. Maize inbreds 178 and 9782 were screened from over 400 maize inbred lines with extreme tolerance and sensitivity to low-P stress in multiple experiments (Zhang et al., 2008; Lin et al., 2013; Zhang L. et al., 2014). Plant growth conditions and data score were the same as those described previously (Zhang L. et al., 2014). In addition, eight teosinte lines, including three each from *Zea perennis*, *Zea mexicana*, and *Zea huehuetenangensis*, two from *Zea nicaraguensis*, and three from *Zea parviglumis*, were used for the analysis of nucleotide diversity and molecular evolution (Supplementary Table S1).

### DNA Sequencing

Genomic DNA was extracted from seedlings at the three-leaf stage using the cetyltrimethyl ammonium bromide (CTAB) method. Sequences of *ZmARF31* (GRMZM2G023813), including the 5′- and 3′-untranslated regions (UTRs), were obtained from 331 maize inbreds and eight teosinte lines by designing three pairs of primers using Primer Premier 5 (Clarke and Warwick, 2001; Supplementary Table S2). PCR was performed as described in the manufacturer’s instruction by using high-fidelity polymerase KOD FX Neo (Toyobo). PCR products from the maize lines and the eight teosinte lines were purified and sequenced directly using ABI 3730 sequencer. For ambiguous chromatograms, the products were re-sequenced in the reverse direction, or the DNA was cloned in pEASY-Blunt Cloning Vector (TransGen Biotech), and sequencing was repeated.

### Analysis of Natural Variation, LD, and Molecular Evolution among Maize Germplasm

The *ZmARF31* polymorphisms among the 331 maize inbreds and eight teosinte lines were detected by conducting multiple sequence alignments by using ClustalX v2.0.11 (Thompson et al., 1997) and Muscle (Edgar, 2004). The confirmed sequences were then improved manually by using BioEdit (Hall, 1999). The genetic diversity of average pairwise nucleotide difference per site (-π) was measured using DnaSP v5.0 (Librado and Rozas, 2009). The LD patterns of *ZmARF31* among maize and teosinte were characterized by calculating *r*^2^ values by using polymorphic sites, including SNPs and InDels, with minor allele frequency (MAF) ≥ 0.05 in TASSEL v3.0 (Bradbury et al., 2007). The LD plot was generated in Haploview (Barrett et al., 2005). The LD decay was measured by averaging *r*^2^ values over a distance of 100 bp and plotting the values against distance.

The evolutionary pressure in *ZmARF31* was further investigated using Tajima’s *D* statistics (Tajima, 1989) and Fu and Li’s test (Fu and Li, 1993). For Fu and Li’s test, *Z. perennis* was used as the outgroup species. A phylogenetic tree including six representative maize inbred lines from different heterotic groups, eight teosinte lines (Supplementary Table S1), and representative monocot and dicot plants (*Sorghum bicolor*: Sb06g033970; *O. sativa:* Os04g59430; *Arabidopsis thaliana*: At1g77850; *Populus trichocarpa*: POPTR 0848s00200g), was generated using the neighbor-joining method in MEGA v6.0 (Tamura et al., 2013). The robustness of the constructed phylogenetic tree was tested using 1,000 bootstrap repetitions.

### Natural Variation and Haplotypes within *ZmARF31* Gene Associated with P-Deficiency-Tolerance Traits

The association between SNPs or InDels in *ZmARF31* gene and the 11 tested traits for low-P tolerance was calculated using TASSEL v3.0 (Bradbury et al., 2007) in three statistical models, i.e., a GLM, GLM with a Q matrix indicative of population structure (GLM + Q), and a mixed linear model (MLM) incorporating both population structure and kinship (K). MLM, including Q and K matrices was considered to be effective for controlling false positives in the association analysis (Liu et al., 2013). The population structure (Q) and relative K were calculated as described by Yang et al. (2011) for the association mapping panel (Li et al., 2013) with the 331 diverse maize inbreds. The threshold of *P* < 0.01 was used for the candidate gene-based association analysis, as described by Liu et al. (2013) and Sosso et al. (2015). As described by Zhang D. et al. (2014), haplotype analysis was conducted using SNPs and InDels located within LD blocks and significantly associated with the tested traits, in order to develop valuable markers for molecular marker assisted breeding. The effects were evaluated in R by ANOVA.

### Validation of Favorable Alleles in Maize RILs

The RIL population (178 × 9782), including 196 lines, was used to detect allele variation in *ZmARF31* for low-P tolerance. Two pairs of special primers, designed based on the flanking sequences of the two significant loci (SNP and InDel), were used to distinguish the presence of favorable alleles in *ZmARF31* by using PCR. The primers are listed in Supplementary Table S2. PCR was performed in 25 μL reactions containing 2.5 μL buffer, 2.5 μL MgCl_2_ (25 mM), 4.0 μL dNTP (2.5 mM), 0.2 μL Taq polymerase (5 U/μL), 1 μL template DNA (approximately 100 ng/μL), and 0.5 μM primers. The PCR conditions were as follows: 94°C for 3 min; 35 cycles at 94°C for 30 s, 58°C for 30 s, 72°C for 30 s, and 72°C for 10 min. The genotypes of the InDel locus were detected based on the amplification products separated on 1.5% agarose gels, stained with ethidium bromide and visualized under UV light, in an electrophoresis systems (Bio-Rad). Two alleles of the SNP locus were distinguished by performing high-resolution melting (HRM) curve (Liew et al., 2004) by using SsoFast^TM^ EvaGreen^®^ supermix (Bio-Rad) and Bio-Rad CFX96 detection system. The reaction volume and cycling conditions for HRM were as per the manufacturer’s instructions, and HRM curves were analyzed using the manufacturer’s software. The *t*-test was used to detect the correlation between allele variation and investigated traits combined with SNP/InDel genotyping and phenotypic data of the RIL population. The favorable alleles containing improved low-P tolerant traits and increased tolerance to P starvation were then identified as described by Liu et al. (2013) and Meihls et al. (2013).

### RNA Extraction and Quantitative Real-Time Reverse Transcription PCR

The two parents of the RIL population, maize inbred lines 178 and 9782, were used to perform expression pattern analysis. Briefly, the seeds of 178 and 9782 were sown on a filter paper saturated with distilled water and germinated at 28°C for 3 days in an incubator. Subsequently, uniformly grown seedlings were transplanted to an aerated complete nutrient solution with 1 mmol/L NH_4_H_2_PO_4_ (PA) in a phytotron to eliminate other influencing factors. At the three-leaf stage, plant seedlings were assigned to PA and NPA conditions. Roots and leaves were separately collected from the maize inbred lines under PA and NPA conditions at 0, 6, 12, 24, 48, and 72 h. Total RNA was isolated from each sample by using TRIZOL reagent (Invitrogen) and RNase-free DNase (Takara). Next, iScript cDNA synthesis kit (Bio-Rad) was used to synthesize cDNA by using 1 mg total RNA from each sample. Subsequently, quantitative real-time reverse transcription PCR (qRT-PCR) was performed using the SsoFast^TM^ EvaGreen^®^ supermix (Bio-Rad), and the expression of housekeeping gene *18S* was used as an internal control. Three replicates were used to calculate the average and SD of expression levels by using the 2^-ΔΔCT^ method (Livak and Schmittgen, 2001) for each sample.

### Subcellular Localization of ZmARF31 Protein

The protein localization of *ZmARF31* gene was determined by conducting subcellular localization analysis in tobacco leaves by using transient transformation. The coding region of *ZmARF31* gene without the stop codon (TAA) was amplified using the specific primers (Supplementary Table S2). The fragment was fused to the N-terminus of enhanced green fluorescent protein (eGFP), which was ligated to pCAMBIA2300 by recombination by using the ClonExpress^®^ II system (Vazyme). A CaMV35S:ZmARF31-eGFP construct was used to assess protein localization. PCXSN2RFP vector, including a nuclear location signal (NLS), was used as a control. The loaded vector pCAMBIA2300-P*_35S_*:ZmARF31-eGFP and control vector were transformed into *Agrobacterium tumefaciens* strain GV3101. Tobacco (*Nicotiana benthamiana*) leaves were used for agroinfiltration by using the transformed GV3101, as described by Yu et al. (2015). The fluorescent signals were detected and images were acquired using an A1R-si laser scanning confocal microscope (LSCM, Nikon, Japan).

## Results

### Phenotypic Variation, Nucleotide Variation, and Molecular Evolution in Maize Germplasm

ANOVA revealed significant phenotypic variation for all the tested traits among the 331 maize inbred lines at *P* < 0.01; and descriptive statistics exhibited a large range of phenotypic variation for each trait (Supplementary Table S3). All the traits were significantly influenced by P starvation, which could be used for further genetic analysis. The natural variation within *ZmARF31* gene was detected by determining the levels of nucleotide diversity in *ZmARF31* by using 331 maize lines and eight teosinte lines. The full-length sequence of *ZmARF31* is 2,148 bp long, which contained five regions (214–1,002 bp in length; **Table 1**). There were 44 polymorphic loci identified with MAF ≥ 0.05 (see Supplementary Table S4 for detailed information). Across the tested 331 maize lines, 30 SNPs and 14 InDels were identified over all the amplicons, with one SNP/InDel every 72/153 bp. The 30 SNPs contained 19 (63%) transitions and 11 (37%) transversions. Comparison of genetic variation across different gene regions suggested that introns contained higher sequence diversity (1.8 polymorphisms/100 bp) than exons (1.1 polymorphisms/100 bp), whereas UTRs showed the highest sequence diversity (4.6 polymorphisms/100 bp).

**Table 1:** Summary of natural variation and neutrality test of *ZmARF31* in maize and teosinte.

Regions	5′-UTR	Exon 1	Intron	Exon 2	3′-UTR	Total
π (× 10^-3^)^a^	4.82	3.52	6.49	4.16	12.36	5.17
Tajima’s *D*_m_	-2.73***	-1.34^ns^	1.36^ns^	-0.84^ns^	-1.46^ns^	-1.96*
Tajima’s *D*_t_	-0.97^ns^	-0.36^ns^	0.21^ns^	-0.63^ns^	-0.77^ns^	-0.52^ns^
Fu and Li’s *D*	-6.79**	-2.63*	0.61^ns^	-4.68**	-3.67**	-6.78**
Fu and Li’s *F*	-5.81**	-2.33*	1.01^ns^	-3.86**	-3.21**	-5.07**
π_m_/π_t_	0.21	0.78	0.19	0.80	0.38	0.36
Length (bp)^b^	230	1002	214	387	315	2148

The Fu and Li’s test were conducted with *Zea perennis* as the outgroup species.

*P < 0.05; **P < 0.02; ***P < 0.01.

*^a^The number of nucleotide differences per site between two randomly chosen sequences.*

*^b^The length of sequence based on maize B73 RefGen_v3.*

*ns, not significant; m, maize; t, teosinte; π, nucleotide diversity; UTR, untranslated region.*

**FIGURE 1:**
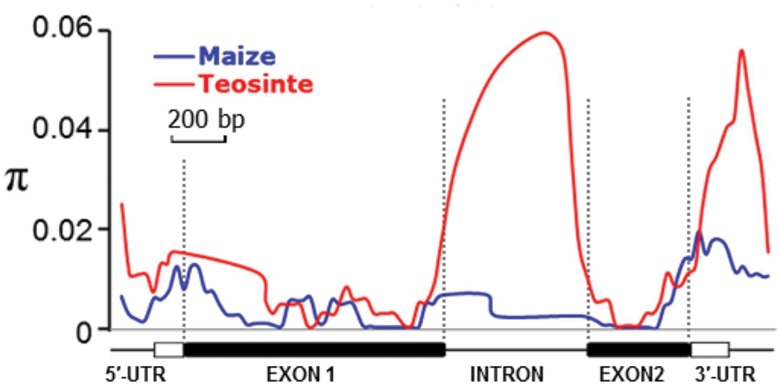
**Nucleotide diversity (π) of *ZmARF31* in maize and teosinte.** A total of 331 maize inbreds and eight teosinte lines were used in sliding window analysis, with window length of 100 sites and step size of 25 sites.

Nucleotide diversity analysis using sliding windows indicated that natural variants in *ZmARF31* were not evenly distributed in either maize or teosinte, and coding regions (exons 1 and 2) contained less genetic variation (**Figure 1**; **Table 1**). Although the 3′-UTR showed the highest nucleotide diversity (π = 12.36 × 10^-3^), exon 1 showed the lowest nucleotide diversity (π = 3.52 × 10^-3^) in maize. The nucleotide diversity ratio (maize/teosinte) was 36%, ranging from 19% (intron) to 80% (exon), indicating that the maize inbred lines have lower genetic variation (**Figure 1**; **Table 1**).

Tajima’s *D* test and Fu and Li’s test for different gene regions were used to understand selection mechanism of *ZmARF31* during maize domestication and improvement. Significant negative Tajima’s *D* values over the entire sequence in maize were consistent with those of Fu and Li’s statistics, suggesting the existence of purifying selection in *ZmARF31* (**Table 1**). Tajima’s *D* test indicated that, except for introns with a positive value, all other regions had negative values in both maize and teosinte. However, the 5′-UTR had significant negative value in maize, indicating that purifying selection and/or population size expansion had occurred in this region. As an additional statistic, Fu and Li’s test using the outgroup species of *Z. perennis* indicated the presence of significant negative values for all genic regions, excluding some non-significant positive values in the introns (**Table 1**). Fu and Li’s test also revealed the signature of purifying selection in some regions of *ZmARF31*.

**FIGURE 2:**
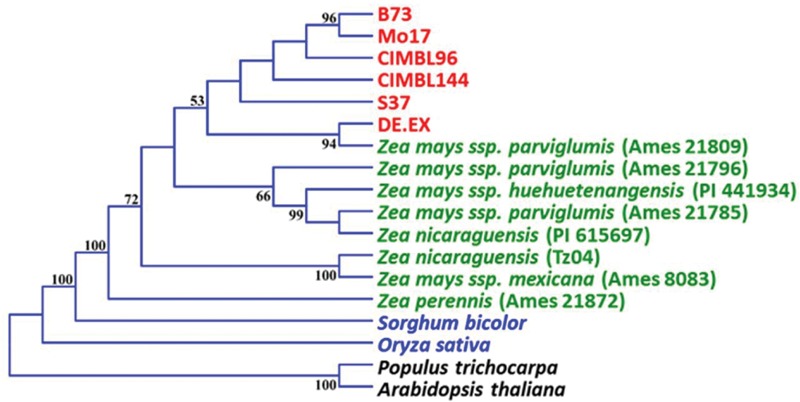
**Phylogenetic analysis of *ZmARF31* in six maize inbred lines, eight teosinte lines and representative monocot and dicot plants.** Numbers on the branches are percentages based on 1,000 bootstrap repetitions, bootstrap values >50% are given.

A phylogenetic tree was constructed for elite maize and teosinte accessions (Supplementary Table S1) using the DNA sequences of *ZmARF31* gene. Under the assumption of only a single favorable haplotype at the neutral loci fixed by past selection, maize sequences would form a single clade during domestication as expected in a previous phylogenetic analysis (Li et al., 2010a). Our phylogenetic analysis showed that maize and teosinte were further combined with other monocot and dicot plants into a well-supported considerably larger clade (**Figure 2**). In particular, all maize inbreds were clustered into a subclade along with their wild ancestor, *Zea mays* ssp. *parviglumis*; which was consistent with the expectation of selective pressure analysis.

### *ZmARF31* Polymorphisms and Haplotypes Associated with P-Deficiency-Tolerance Traits

**FIGURE 3:**
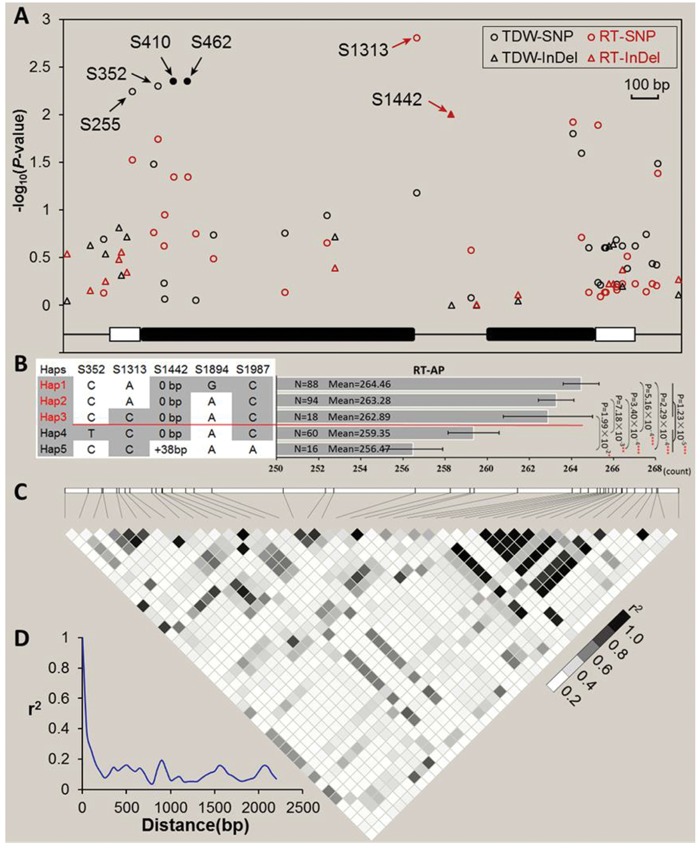
**Natural variation and haplotype within *ZmARF31* are associated with P-deficiency-tolerance traits. (A)** Natural variation in *ZmARF31* associated with total dry weight (TDW) and root tips (RT) under P-applied condition. Each circle and triangle represents a polymorphic SNP and InDel site, respectively. The *P* value is shown on a -log_10_ scale. A schematic diagram of the entire gene structure is presented as the *x*-axis, including white and black boxes showing as UTRs and exons, respectively. **(B)** Effect of haplotypes consisting of five significant loci on RT in 331 maize inbreds. Hap, haplotype; Hap1–Hap5 with MAF ≥ 0.05 were used for the statistic test. **(C)** The pattern of pairwise LD of DNA polymorphisms (MAF ≥ 0.05) in *ZmARF31*. The shaded color reflects the level of LD (*r*^2^). **(D)** LD decay in the DNA sequence of *ZmARF31* in maize. Measurement was performed by averaging *r*^2^ values of allele frequencies over a distance of 100 bp and plotting the values against distance (bp).

LD patterns showed discrete blocks in various regions of *ZmARF31* (**Figure 3C**). An LD block was observed at the 5′-UTR, where the signature of purifying selection were detected. However, a larger LD block was observed in the 3′-UTR that also showed the most abundant diversity (**Table 1**; **Figure 3C**). Plots of *r*^2^, representing of LD level, indicated that LD declines to 0.1 within 1 kb in maize lines (**Figure 3D**), which is considerably faster than the average of the whole maize genome (Gore et al., 2009).

The association between natural variation in *ZmARF31* and low P tolerance was investigated using three statistical models. Of the 1,936 possible combinations (44 sites × 11 traits × 2 treatments × 2 years), 220, including 146 under PA and 74 under NPA conditions, were significant at *P* ≤ 0.05. Using GLM, GLM + Q, and MLM (Q + K), 91, 73, and 56 associations were significant at *P* ≤ 0.05, 30, 14, and 9 of which were significant at *P* ≤ 0.01, respectively (**Table 2**). Among the 44 polymorphic sites, 18 (including two InDels) were found to be significantly associated at *P* ≤ 0.01 with at least one of the P-deficiency-tolerance traits (**Table 2**; Supplementary Table S5). Four identified SNPs (S255, S352, S410, and S462) were significantly associated with TDW as revealed by all the three models, of which S410 and S462 were located in the complete LD block. Further, SNP S1313 and InDel S1442 in the intron region were significantly associated with RT, explaining 3.6 and 2.5% of phenotypic variation, respectively (**Figures 3A,C**; **Table 2**). Detailed information on the location, genotype, frequency, and statistical value for each site is presented in Supplementary Table S5.

**Table 2:** Associations between the natural variations within *ZmARF31* and phosphorus-deficiency-tolerance traits.

Site	SNP/InDel^a^	MAF	GLM			GLM + Q			MLM		
			No. sig	Range *P* (×10^-3^)	Range *R*^2^ (%)	No. sig	Range *P* (×10^-3^)	Range *R*^2^ (%)	No. sig	Range *P* (×10^-3^)	Range *R*^2^ (%)
S255	T/C	0.25	5	0.99–9.79	2.22–3.26	2	5.19–6.26	2.77–2.90	1	5.73	2.91
S335	A/G	0.35	1	5.5	2.32						
S352	T/C	0.24	4	2.01–6.93	2.45–2.87	2	3.01–4.51	3.0–3.25	2	5.00–6.73	2.78–3.00
S410	T/G	0.48	2	9.12–9.38	2.05–2.28	1	5.31	2.88	1	4.48	3.08
S462	A/G	0.48	2	9.12–9.38	2.05–2.28	1	5.31	2.88	1	4.48	3.08
S492	T/C	0.11	1	5.68	2.30	1	8.57	2.19			
S823	T/C	0.42	1	4.06	2.48						
S980	A/G	0.35	1	6.16	2.26						
S1313	A/C	0.37	1	1.12	3.49	2	1.53–7.94	2.24–3.60	1	3.80	3.14
S1442	0/38	0.08	2	4.90–5.00	2.49–2.74	1	9.95	2.5			
S1535	0/4	0.17	2	3.73–8.95	2.29–2.81						
S1894	A/G	0.29	1	8.59	2.31						
S1926	C/G	0.46				2	0.29–1.86	3.44–4.53	2	0.90–2.66	3.37–4.09
S1987	A/C	0.07	2	4.78–8.70	2.07–2.66						
S1998	T/C	0.12	1	5.81	2.56						
S2191	T/C	0.11	1	6.24	2.52						
S2207	C/G	0.11	1	6.03	2.54						
S2211	A/G	0.26	2	1.31–9.37	2.27	2	2.20–7.13	2.67–3.27	1	6.20	2.77
Total	18		30	0.99–9.79	2.05–3.49	14	0.29–9.95	2.19–3.60	9	0.90–6.73	2.77–4.09

*^a^The letters indicate nucleotide polymorphisms and the numbers indicate the inserted or deleted nucleotides. Underlined letters and numbers represent the minor alleles.*

*No. sig, the total number of significant associations detected at *P* < 0.01; MAF, minor allele frequency; *R*^2^, explained phenotype variation; SNP, single nucleotide polymorphism; InDel, insertion and/or deletion; GLM, general linear model; MLM, mixed linear model; Q, Q matrix of population structure.*

The effects of gene polymorphisms on root trait response to low P stress were determined by analyzing haplotypes with significant SNPs/InDels in *ZmARF31*. Five SNPs/InDels (MAF ≥ 0.05) with moderate to strong LD levels that were significantly associated with RT under PA condition (*P* ≤ 0.01) were evaluated, and eight haplotypes were identified (**Figure 3B**). Of them, five haplotypes (Hap1–Hap5) with an MAF of ≥5% were analyzed on a P-deficiency-tolerance trait, RT. Significant differences in RT were found between pairwise haplotypic comparisons (hap1 vs. hap 4, hap2 vs. hap4, hap2 vs. hap5, hap1 vs. hap5, and hap3 vs. hap5) at *P* < 0.05. The haplotypes with more favorable alleles (Hap1, Hap2, and Hap3) showed significantly (*P* < 1.23 × 10^-5^) higher RT than those with less favorable alleles (Hap4 and Hap5; **Figure 3B**). Haplotypes had higher explained phenotypic variation for RT (7.65%) compared to single loci (from 2.31 to 3.60%). Another gene region, which was at the extremely strong LD level (*r*^2^ > 0.95), contained five SNPs (S1953, S2012, S2018, S2078, and S2127) and two InDels (S2029 and S2045) and captured only two haplotypes with MAF ≥ 0.05, showed significant effect on TDW under the NPA condition (*P* < 0.05). Moreover, haplotypes from the SNPs and InDels that were significantly associated with other tested traits were also analyzed without considering LD. At *P* < 0.01, four informative haplotypes with MAF ≥ 0.05, consisting of five SNPs significant for NL (S255, S352, S410, S462, and S2211) and one nearby SNP S1998 (or nearby SNP 492), showed significant haplotypic effect on the target trait, NL. Four informative haplotypes from six SNPs and one InDel (S255, S335, S410, S823, S1535, S1926, and S2191) were significantly associated with NL and RD. Five haplotypes consisting of three SNPs and two InDels (S492, S1313, S1442, S1535, and S1987) had significant effect on LLR. The combinations of the loci that were significantly associated with target traits explained higher proportions of phenotypic variation than single loci and also represented functional haplotypes.

### Favorable/Tolerant Alleles of *ZmARF31* Associated with Improved P Tolerance in Maize

**FIGURE 4:**
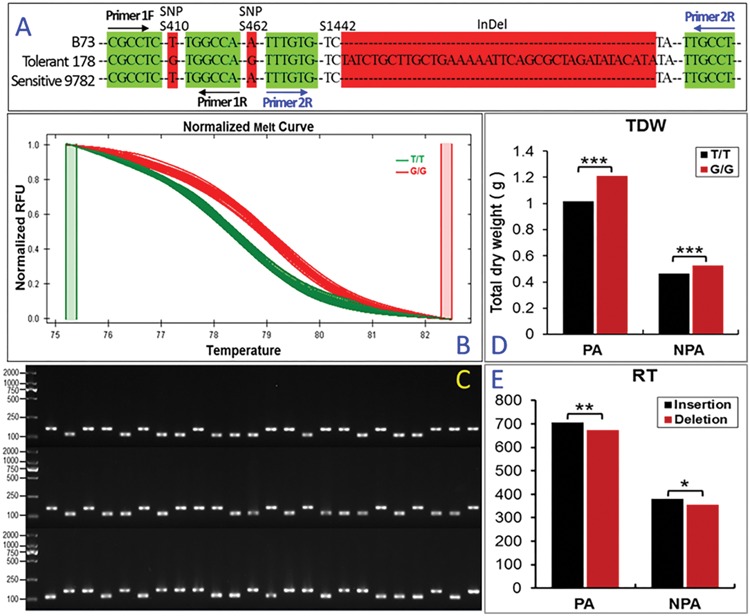
**The favorable allele of *ZmARF31* improves maize phosphorus tolerance. (A)** DNA polymorphisms of three sites in *ZmARF31* among B73, 178 and 9782. SNPs S410 and S462, and InDel S1442 that were significantly associated with phosphorus-deficiency-tolerance traits are shaded in red. The location of PCR primers used for DNA amplification of S410 and S1442 are shaded in green by arrows. **(B)** Normalized high-resolution melting curve to distinguish genotypes of SNP S410 in the RIL population. Green and red curves indicate allelic SNPs, T and G. **(C)** Length polymorphism amplicons of InDel S1442 in the RIL population. Upper and lower lanes indicate allelic sequence loci, insertion with 38 bp and deletion. **(D,E)** The effect of the favorable allele in S410 on TDW and the favorable allele in S1442 on RT in the RIL population. The traits of the two groups with homozygous genotypes was assessed using *t*-test (*P < 0.05, **P < 0.01, ***P < 0.001). RIL, recombination inbred line; TDW, total dry weight; RT, root tips; PA, P-applied condition; NPA, non-P-applied condition.

The genetic effect of natural variations within *ZmARF31* on low-P tolerance in maize was elucidated using an RIL population with 196 lines and its two parental lines. The alleles from significant loci in the tolerant inbred 178 and sensitive inbred 9782 were considered to be favorable/tolerant and inferior/sensitive, respectively. The DNA polymorphisms at significant SNPs S410 (T/G) and S462 (A/G) associated with TDW and InDel S1442 (0/38 bp) associated with RT were detected (**Figure 4A**). The allele “G” at SNP locus S410 and the large insert fragment with 38 bp located in the intron region at S1442 were found in the low-P-tolerant inbred 178 (**Figure 4A**). Two pairs of special primers, designed based on the flanking sequences at the two significant loci (**Figure 4A**), were used to successfully distinguish the presence of favorable alleles in *ZmARF31* in the RIL population (**Figures 4B,C**). For S410 (T/G), two line groups with alleles “T” and “G” in the RIL population were identified using the normalized melting curve of HRM (**Figure 4B**). For S1442 (0/38 bp), “insertion” and “deletion” with 38 bp were distinguished and revealed through a fragment length polymorphism in the RIL population (**Figure 4C**).

The genetic effects of SNP S410 (T/G) and InDel S1442 (0/38 bp) on the associated traits TDW and RT were examined. A significant difference in TDW between the line groups with homozygous allelic SNP T/T and G/G was found under both PA and NPA conditions (**Figure 4D**). The line group with SNP G/G had higher mean value than the group with SNP T/T, implying that the SNP G/G was favorable/tolerant. The low-P-tolerant inbred 178 had favorable/tolerant allele GG at SNP S410 (T/G), while the low-P-sensitive inbred 9782 had allele TT. A significant difference in RT was observed between the line groups with homozygous allelic insertions and deletions (**Figure 4E**). As expected, compared with NPA treatment, the RIL population showed substantially increased mean values for both traits under PA condition. Further, the lines with favorable/tolerant allele insertion were more tolerant to P starvation than those with inferior/sensitive allele deletions. Thus, natural variation at the tested SNP and InDel in *ZmARF31* contributed to the improved low-P tolerance in maize.

### Expression Analysis of *ZmARF31* Gene in Maize

**FIGURE 5:**
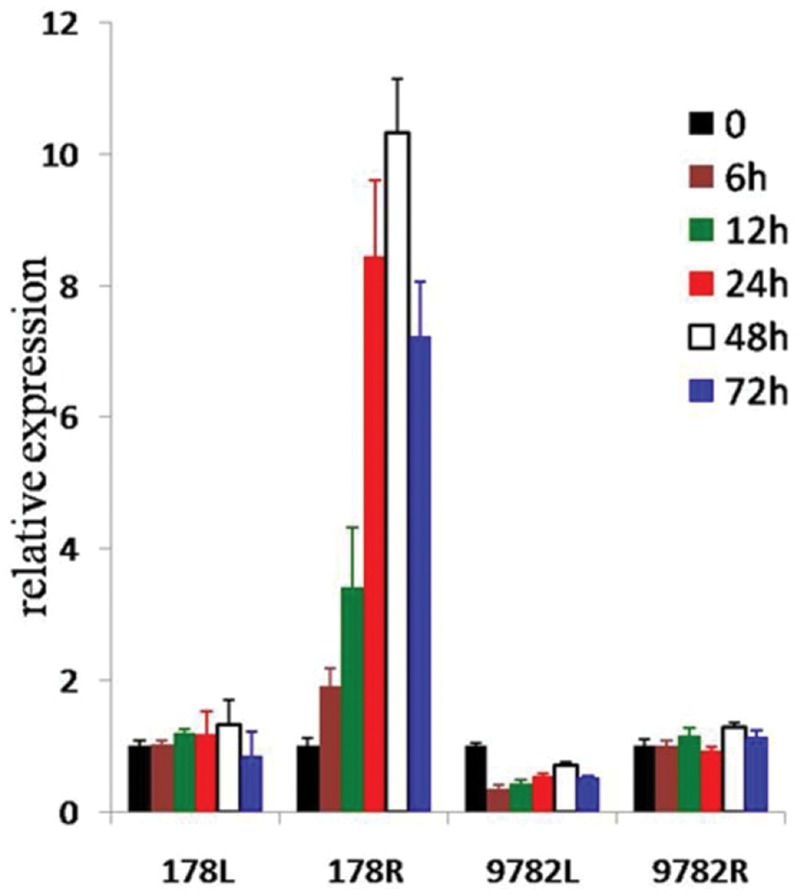
**Expression analysis of *ZmARF31* in low-phosphorus-tolerant and low-phosphorus-sensitive inbreds, 178 and 9782.** Low phosphorus stress was conducted at seedling stage with treatments of 0, 12, 24, 48, and 72 h. L, leaves; R, roots.

The expression patterns of *ZmARF31* under PA and NPA conditions were determined using qRT-PCR for leaves and roots collected from the low-P-tolerant and sensitive inbreds, 178 and 9782. *ZmARF31* gene showed different expression patterns in leaves and roots from two maize inbreds (**Figure 5**). The highest relative expression levels were observed in the roots of low-P-tolerant inbred 178, which were gradually and constitutively induced after NPA treatment, with an increased expression of over 10-fold at 48 h. However, stable mRNA levels were observed for the leaves of 178 across different treatment time points. This finding was considerably similar to the expression trend observed for the roots of low-P-sensitive inbred 9782, where the expression levels of *ZmARF31* were lower in the leaves than those in the roots. These results suggested that *ZmARF31* was possibly involved in maize low-P tolerance.

### Subcellular Localization of ZmARF31 Protein

**FIGURE 6:**
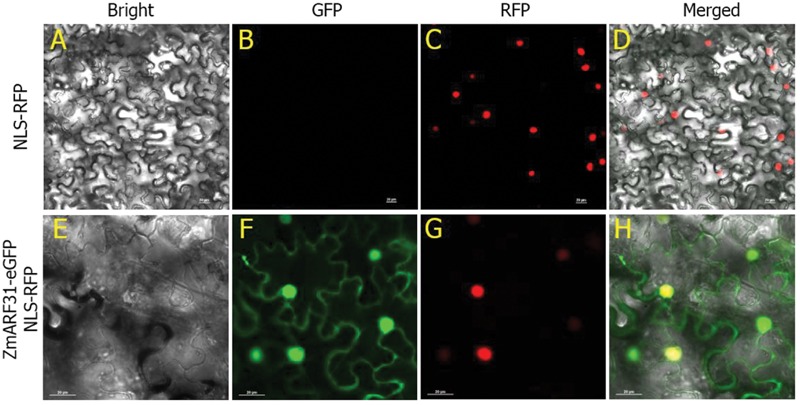
**Subcellular localization of the ZmARF31 protein in *N. benthamiana* leaves.** NLS-RFP and ZmARF31-eGFP were driven by CaMV35S promoter and transiently expressed in *N. benthamiana* leaves. **(A–D)** Tobacco leaf transformed by CaMV35S:NLS-RFP as a control. **(E–H)** ZmARF31-eGFP targeted to the cell nucleus and cytoplasm in tobacco leaf. The images are shown in bright-field **(A,E)**, the GFP fluorescence (green) only **(B,F)**, the red fluorescent protein (RFP) fluorescence (red) only **(C,G)**, and merged **(D,H)**, respectively. Scale bars, 20 μm.

The subcellular localization of ZmARF31 protein was determined by introducing the loaded vector pCAMBIA2300-P*_35S_*:ZmARF31-eGFP and control vector PCXSN2RFP into tobacco leaves using transient transformation. LSCM revealed strong red fluorescence signals only in the nucleus of the tobacco leaves transformed by CaMV35S:NLS-RFP as the control (**Figures 6A–D**), whereas the fusion protein, ZmARF31-eGFP, showed green fluorescence signals in both nucleus and cytoplasm (**Figures 6E–H**). This result confirmed the prediction by the CELLO v.2.5 program (Yu et al., 2006) that ZmARF31, a TF, was expressed in both nuclear and cytoplasmic compartments of tobacco leaves.

## Discussion

Maize has been exploited as a model organism for basic and applied studies of domestication and genetic improvement owing to its massive phenotypic variation and genetic diversity (Strable and Scanlon, 2009; Yan et al., 2011; Liu et al., 2013). The average nucleotide polymorphism in the two maize lines is even greater than the divergence between humans and chimpanzees (Buckler et al., 2006). The high level of natural variation results mainly from the substantial genetic diversity in its ancestor teosinte, without serious domestication bottleneck (Tian et al., 2009). Previous studies revealed an average frequency of one SNP per 104 bp on chromosome 1 (Tenaillon et al., 2001) and one SNP per 61 bp in 18 maize genes identified with 36 elite maize inbreds (Ching et al., 2002). A high level of genetic diversity in *ZmARF31* was also found in this study, with one polymorphic site (either SNP or InDel) every 49 bp. Different levels of nucleotide polymorphism detected across different studies might be attributed to gene properties in specific plant species and population size (Yu et al., 2013). The structural heterogeneity in *ZmARF31* (**Table 1**) was diverse compared to that in *PSY1* (Fu et al., 2010), suggesting that both SNPs and InDels might be important to generate novel variations during species evolution.

The target gene that had been subjected to selective constraints during maize domestication showed the signature of evolutionary pressure in its DNA sequence polymorphism (Wang et al., 2005). Both Tajima’s *D* and Fu and Li’s tests showed the presence of purifying selection in *ZmARF31*, especially in its 5′-UTR. However, some gene regions showed no significant selection signals, suggesting that artificial selection does not affect the entire gene, which is in agreement with previous studies (Wang et al., 2005; Li et al., 2010b). The reduction in relative genetic diversity of the 5′-UTR in *ZmARF31* is lower than that observed for selective pressure genes such as *tb1*, *ts2*, *d8*, and *zagl1* (Clark et al., 2004; Tenaillon et al., 2004), but higher than that in the transposable element of *ZmCCT*, a domestication gene that is activated in response to photoperiod (Yang et al., 2013). This implies the presence of selection in the 5′-UTR. Further, phylogenetic analysis indicated that maize and teosinte are clustered into separate clades, respectively, which supports that signature of evolutionary pressure must be present in *ZmARF31*. Taken together, our results and previous findings provide strong evidence for selection in some genes during maize domestication, such as *ZmARF31* for low-P stress, *tga1* for teosinte glume architecture (Wang et al., 2005) and *tb1* for teosinte branching (Studer et al., 2011).

The level of LD in a genome is a criterion on which resolution and feasibility for association mapping of complex traits are established (Clark et al., 2004). The variation in LD decay indicates the difference in relative recombination rates and population genetic factors (Clark et al., 2004; Yan et al., 2011). The overall LD decay of *ZmARF31* revealed in this study was similar to that reported previously, i.e., within a distance of 500–1,500 bp, depending on population size and tested genes (Tenaillon et al., 2001; Yu et al., 2013). Different rates in LD decay in candidate genes, including *ZmARF31*, compared with LD decays at a distance within 2 kb for the entire maize genome (Gore et al., 2009), indicates a non-uniform genome evolution and selective sweep in maize. Because of different LD decay rates in *ZmARF31* among diverse maize inbreds, nucleotide variations associated with low-P-tolerance traits could be identified using different models, with 30, 14, and 9 significant associations detected using GLM, GLM + Q, and MLM models, respectively. However, spurious associations that might be contributed by population structure and cryptic individual relatedness in the given panel are a major concern for GLM model. In general, Anderson–Darling test and MLM could be a good complement to current popular association methods, which can eliminate the excess of low *P* values for most traits (Yang et al., 2014). In our study, seven overlapped natural variations were revealed when GLM and MLM were used to control false positives. Further, MLM overcompensates for population structure along with familial relatedness, leading to an increase of false negative rate (Yang et al., 2014). For example, we validated InDel S1442 (0/38 bp) that was significantly associated with RT in the RIL population with GLM and GLM + Q models, but not with MLM model. A positive genetic effect on RT with 38 bp insertion as favorable/tolerant allele was identified, indicating that InDel S1442 showed robust association with the target trait (**Figure 3A**). Furthermore, HRM-based verification for non-synonymous SNP mutation S410 (T/G) revealed that favorable/tolerant allele G/G had a positive genetic effect on TDW (**Figures 4B,D**). This is similar to a previous report on co-segregation of the *ZmDREB2.7* tolerant allele with improved drought tolerance (Liu et al., 2013), where plants homozygous for the favorable/tolerant alleles of *ZmDREB2.7* were more tolerant to drought stress than those homozygous for the inferior/sensitive alleles.

Further haplotype analysis with five significant variation loci, including InDel S1442, revealed that the haplotypes with more favorable SNPs/InDels had a greater effect on phenotypic variation than single loci (Lu et al., 2010). The expression analysis further confirmed that *ZmARF31* responded to low P stress and affected low-P tolerance in different genotypes, with varied expression levels in root and leaf tissues of extremely tolerant and sensitive inbred lines (**Figure 5**). Compared with low-P-sensitive inbred 9782, a higher expression level was detected in the roots and leaves of low-P-tolerant inbred 178. The results also indicate that the candidate gene association mapping within ZmARF31 gene region might be helpful to mine natural variation associated with low-P stress. As reported by Xing et al. (2015), a rare SNP mutation in Brachytic2 coding sequence moderately reduces plant height and increases yield potential in maize.

To determine the subcellular localization of the functional gene, the target fragment is fused with eGFP/GFP forming a fusion protein, and then an expression system can be used to determine its location based on the protein signal. Subcellular localization with the heterologous system using CaMV35S as a promoter, especially in tobacco leaves or onion epidermal cells is widespread used in different species including maize. For example, photoperiod sensitivity gene in maize, *ZmCCT*, was detected in nucleus by CaMV35S:ZmCCT-GFP introduced into onion epidermal cells (Yang et al., 2013). Tobacco leaves were transiently infiltrated with GV3101 containing vector expressing CaMV35S:VaNAC26-eGFP and the protein encoded by maize gene *NAC26* was located in nucleus (Fang et al., 2016). In our study, the subcellular localization by transient expression in tobacco indicated that the protein encoded by *ZmARF31* was located in both nucleus and cytoplasm, which was different from the general phenomenon that TFs are only located in the nucleus. The difference can be explained by the special biological function of *ZmARF31*. The subcellular distribution of ZmARF31 is regulated by various cellular processes, which might include the degree of phosphorylation during P starvation (Brownawell et al., 2001), time-/dose-/concentration-dependence of ATP (Choi et al., 2012), as well as the presence of isoform, reducing its ability to interact with nuclear-binding sites (Parnell et al., 2015). However, these special cellular processes need to be further examined in maize. In conclusion, natural variation in *ZmARF31* associated with low-P tolerance might allow the elucidation of the molecular mechanism underlying response to low-P stress under various maize genetic backgrounds. The detailed phenotypic and molecular characterization of allele variations in *ZmARF31* will be useful for accelerating improvement of tolerance to abiotic stress and increasing molecular breeding efficiency in maize.

## Author Contributions

YLu and FW conceived the experiments and designed the study. FW, ZL, JX, and LL performed the experiments and analyzed the data. SG and HL provided some maize lines and the RIL population. FW, YLi, and YLu drafted the manuscript, which was reviewed by all authors.

## Conflict of Interest Statement

The authors declare that the research was conducted in the absence of any commercial or financial relationships that could be construed as a potential conflict of interest.
